# Malaria morbidity and mortality in Ebola-affected countries caused by decreased health-care capacity, and the potential effect of mitigation strategies: a modelling analysis

**DOI:** 10.1016/S1473-3099(15)70124-6

**Published:** 2015-04-23

**Authors:** Patrick G T Walker, Michael T White, Jamie T Griffin, Alison Reynolds, Neil M Ferguson, Azra C Ghani

**Affiliations:** MRC Centre for Outbreak Analysis and Modelling, Imperial College London, London, UK

## Abstract

**Background:**

The ongoing Ebola epidemic in parts of west Africa largely overwhelmed health-care systems in 2014, making adequate care for malaria impossible and threatening the gains in malaria control achieved over the past decade. We quantified this additional indirect burden of Ebola virus disease.

**Methods:**

We estimated the number of cases and deaths from malaria in Guinea, Liberia, and Sierra Leone from Demographic and Health Surveys data for malaria prevalence and coverage of malaria interventions before the Ebola outbreak. We then removed the effect of treatment and hospital care to estimate additional cases and deaths from malaria caused by reduced health-care capacity and potential disruption of delivery of insecticide-treated bednets. We modelled the potential effect of emergency mass drug administration in affected areas on malaria cases and health-care demand.

**Findings:**

If malaria care ceased as a result of the Ebola epidemic, untreated cases of malaria would have increased by 45% (95% credible interval 43–49) in Guinea, 88% (83–93) in Sierra Leone, and 140% (135–147) in Liberia in 2014. This increase is equivalent to 3·5 million (95% credible interval 2·6 million to 4·9 million) additional untreated cases, with 10 900 (5700–21 400) additional malaria-attributable deaths. Mass drug administration and distribution of insecticide-treated bednets timed to coincide with the 2015 malaria transmission season could largely mitigate the effect of Ebola virus disease on malaria.

**Interpretation:**

These findings suggest that untreated malaria cases as a result of reduced health-care capacity probably contributed substantially to the morbidity caused by the Ebola crisis. Mass drug administration can be an effective means to mitigate this burden and reduce the number of non-Ebola fever cases within health systems.

**Funding:**

UK Medical Research Council, UK Department for International Development, Bill & Melinda Gates Foundation.

## Introduction

Since the Ebola outbreak in Guinea was first reported to WHO on March 23, 2014, the virus has spread to nine countries, leading to 25 826 cases and 10 704 deaths by April 12, 2015.^1^ Sustained transmission of the virus is occurring in three countries in west Africa: Guinea, Liberia, and Sierra Leone.^2^ The high case fatality ratio of the disease, coupled with high transmission to health-care professionals and low specificity of early symptoms of Ebola virus disease, has placed extraordinary strain on health systems in these countries. As a result, few patients have access to health-care facilities, with many facilities closed. In those still open, fear of the disease has decreased outpatient attendance to as low as 10%.^3^

As a result, the Ebola epidemic in parts of west Africa will probably have a much greater effect than the direct morbidity and mortality effects of the disease alone. The near cessation of malaria control could lead to a resurgence in malaria cases and deaths, reversing progress made over the past decade.^4^ An increase in malaria prevalence will also increase the number of people who have fever-like symptoms, further complicating the identification and treatment of people with Ebola virus disease.

In response to these concerns, the WHO Global Malaria Programme has released guidance on temporary malaria control measures that should be considered.^3^ These measures include strategies to reduce malaria morbidity and mortality and to relieve Ebola assessment services by reducing the prevalence of non-Ebola-related fever in affected areas. One recommendation is to deploy mass administration of long-lasting artemisinin combination treatment drugs not used as first-line treatment. Campaigns would occur for 2–3 months, after which the possibility of extending the campaign could be assessed.^3^ Such a strategy aims to provide rapid protection from malaria in areas where health care is inadequate and to avoid the added risk of Ebola virus infection and the health-care burden associated with treating malaria in clinics. These mass drug administration campaigns are being implemented;^5^ however, the probable effect of such strategies has not been properly assessed.

We estimated the effect that cessation of usual health-care provision for malaria as a result of the Ebola epidemic has had on malaria transmission, case numbers, and deaths. We then assessed the benefit of a mass drug administration campaign initiated in 2015 to reduce malaria-attributable deaths and the burden of malaria-attributable fever on the health systems in the three affected countries.

## Methods

### Estimation of the effect of health-system failure on malaria transmission and prevalence

We used a previously reported model^6^ to model malaria transmission in Guinea, Liberia, and Sierra Leone from 2000, to the start of the Ebola outbreak in March, 2014 (appendix). All model runs were done at the level of the second administrative unit in Sierra Leone (districts) and Liberia (counties) and the first administrative unit in Guinea (regions). The model was fitted to match estimates of parasite prevalence by microscopy in children younger than 5 years, using the proportion of fevers in these children treated with antimalarial drugs and use of insecticide-treated bednets in each of the three countries as input parameters. These data were obtained from the most recent final report of a Demographic Health Survey (DHS) or Malaria Indicator Survey (MIS; appendix). To capture the decrease in malaria transmission that has occurred since 2005, we also incorporated data about use of insecticide-treated bednets from older DHS and MIS surveys, linearly interpolating between these estimates for years in which surveys were not available.

We used the model to estimate the number of clinical malaria cases that would have occurred up to the end of November, 2014, in the absence of the Ebola epidemic, using a previously fitted relation between parasite prevalence and clinical incidence.^6^ We then compared this estimate with a scenario in which health systems no longer provide malaria treatment after 2 months of sustained transmission of Ebola virus based on patient database records published by WHO^1^ (from May, 2014, onwards in Guinea, and July, 2014, onwards in Sierra Leone and Liberia) to estimate the increase in malaria incidence attributable to the ongoing Ebola epidemic in 2014. We estimated the additional number of malaria cases caused by the Ebola epidemic by multiplying the age-stratified case incidence and age-stratified population estimates for each administrative unit. We assessed uncertainty in the estimates by use of Bayesian methods, presented as 95% credible intervals (CrI).

The epidemic probably disrupted the distribution of bednets in affected areas, thereby reducing their effectiveness because nets degrade over time as a result of wear and tear and waning insecticide concentration. We therefore used an existing model of the effect of insecticide-treated bednets^7^ to explore the potential effect of cessation of bednet distribution, comparing a situation in which bednets are replenished every 3 years, with a third of the covered population receiving a net each year and at 20% attrition of net use per year between rounds, with a situation in which bednet distribution ceases as a result of the Ebola epidemic.

### Estimation of the effect of health-systems failure on the likelihood of death after clinical disease

We used a decision tree model to estimate the risk of death from a clinical case of malaria under health systems of differing levels of functioning (appendix). We drew parameters for the decision tree from the country-specific DHS and MIS population surveys, Delphi surveys,^8^ and the World Malaria Report.^9^ We used the model to estimate malaria-attributable mortality in each administrative unit in the presence of a health system with pre-Ebola capacity (using country-specific treatment rates) and in the absence of both clinic or hospital capacity for malaria treatment. We then obtained the additional numbers of malaria-attributable deaths caused by the Ebola epidemic by multiplying mortality by population size, taking into account the age-distribution of the population and of malaria cases in each administrative unit. We calculated uncertainty in the estimates by combining uncertainty in case numbers and uncertainty in parameters in the decision tree model to give approximate 95% CrI.

### Modelling the effect of emergency mass drug administration

We simulated mass drug administration campaigns in 2015 involving either three or six rounds of complete doses of either artesunate–amodiaquine (the recommended first-line treatment in Guinea, Sierra Leone, and Liberia) or the long-acting antimalarial drug dihydroartemisinin–piperaquine, both given at monthly intervals.^10^ We considered three levels of coverage of mass drug administration (30%, 50%, and 70%) of the at-risk population within each administrative unit in each round and we assumed that the coverage between rounds was correlated (eg, the same 30% of individuals received the drug each round while the same 70% received no treatment). We varied the beginning of this campaign between January and September for each country and compared the effect on malaria cases and deaths of an immediate campaign (beginning in January, 2015) with one for which the timing of the campaign was chosen to minimise malaria burden and health-system demand over the whole year (beginning in June, 2015).

### Role of the funding source

The funders of the study had no role in study design, data collection, data analysis, data interpretation, or writing of the report. The corresponding author had full access to all the data in the study and had final responsibility for the decision to submit for publication.

## Results

**Figure 1:**
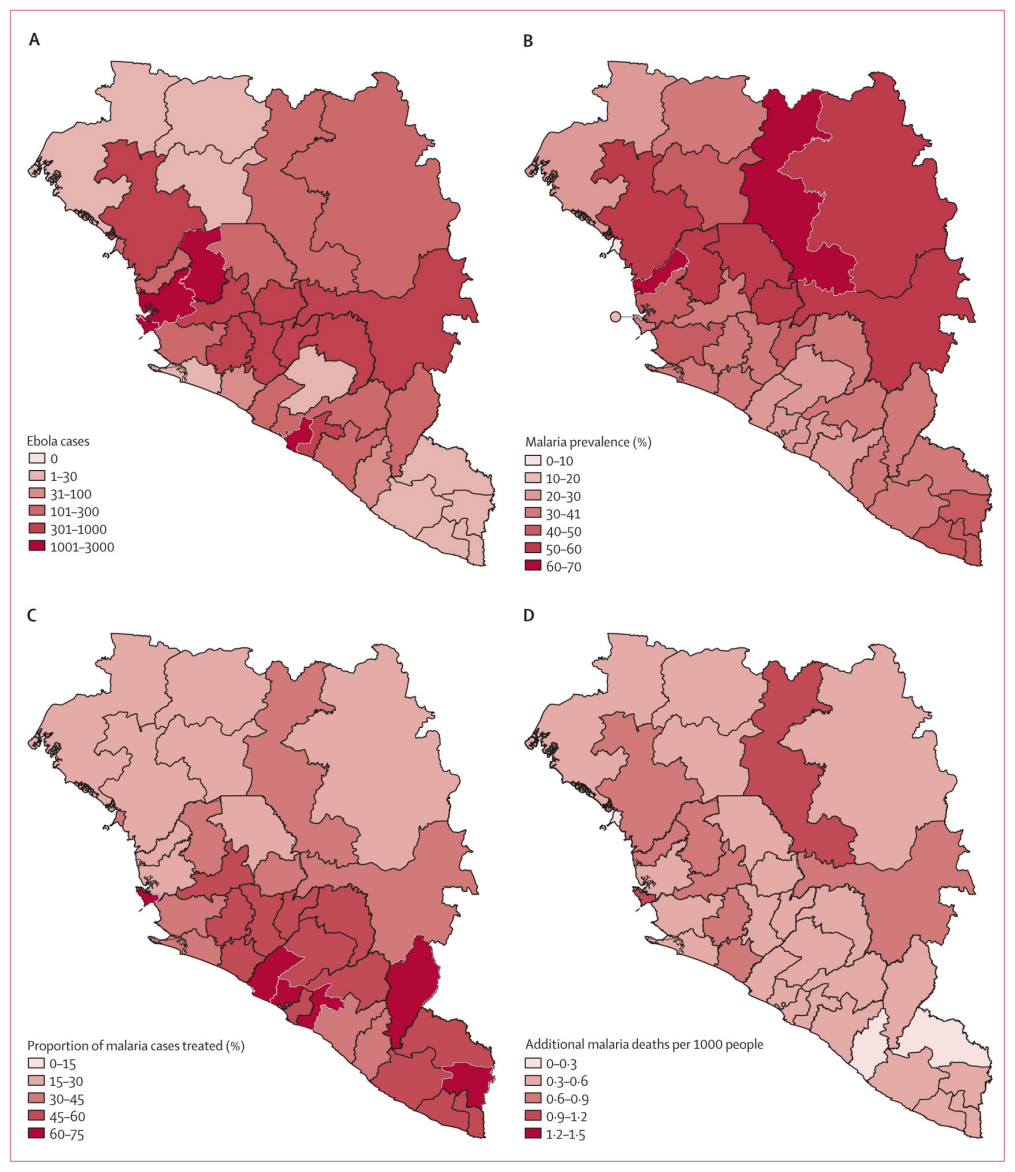
Distribution of Ebola virus disease, malaria prevalence, malaria treatment coverage, and estimated effect of Ebola virus disease on malaria mortality in Guinea, Liberia, and Sierra Leone (A) Cases of Ebola virus disease up to Feb 1, 2015, with data from patient databases except for Liberia from Nov 17, 2014, for which data are from situation reports only.^1^ (B) Prevalence of malaria in children younger than 5 years according to slide microscopy. (C) Proportion of fevers treated with malaria in children younger than 5 years, from both population-based surveys, and our estimates of the additional malaria mortality caused by Ebola virus disease (D).

With the exception of some urban centres such as Conakry in Guinea, malaria is hyperendemic in almost all areas where Ebola virus transmission is sustained (figure 1A), with population-based estimates of parasite prevalence in children younger than 5 years of 43·9% in Guinea, 27·8% in Liberia, and 42·9% in Sierra Leone.^11–13^

**Figure 2:**
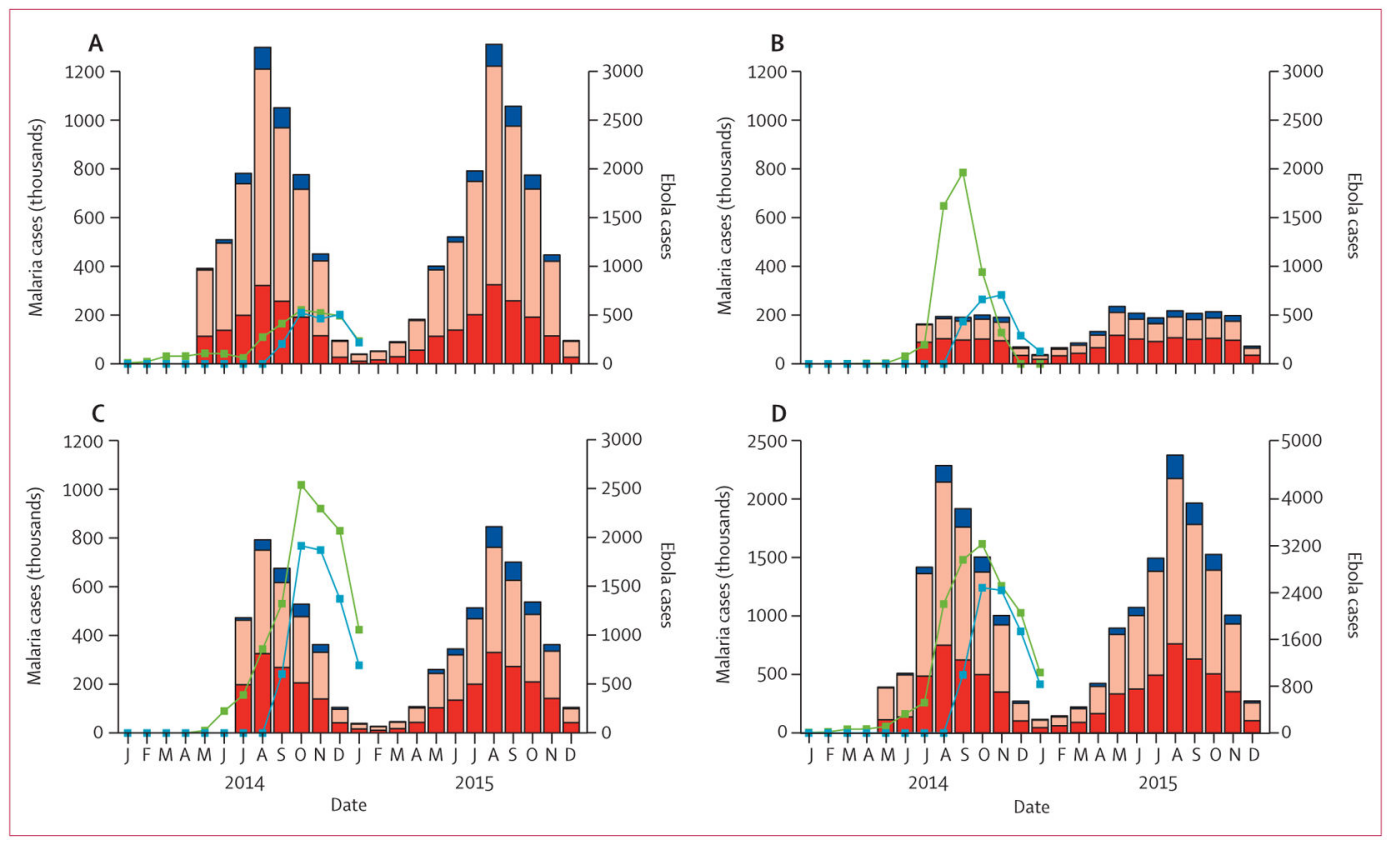
Effect of health-systems failure on incidence of untreated malaria For Guinea (A), Liberia (B), Sierra Leone (C), and the combined total (D). Pink bars show the number of cases untreated and red bars show the number of cases treated when the system is functioning normally, blue bars show additional cases caused by increases in transmission from the additional untreated cases. The green lines show the present status of the Ebola epidemic (probable and confirmed cases from patient databases), the blue lines show Ebola cases from WHO situation reports.

In Guinea and Sierra Leone, where malaria transmission is highly seasonal, the sharp rise in cases of Ebola virus disease in the middle of 2014 was estimated to have coincided with the peak months of malaria transmission. In Liberia, malaria is endemic for most of the year, albeit at lower overall levels than in the Guinea and Sierra Leone (figure 2). Thus, the timing of the Ebola epidemic has probably exacerbated the burden on health systems in these countries and hence had a substantial effect on the number of malaria patients who received treatment. If the Ebola epidemic led to a complete cessation of malaria treatment, we estimated that the number of untreated malaria cases will have increased by 45% (95% CrI 43–49) in Guinea, 88% (83–93) in Sierra Leone, and 140% (135–147) in Liberia in 2014, representing a total of 3·5 million (2·6 million to 4·9 million) additional untreated cases: 1·6 million (1·1 million to 2·4 million) in Guinea, 1·3 million (0·9 million to 1·9 million) in Sierra Leone, and 0·52 million (0·36 million to 0·76 million) in Liberia. This increase translates to an increase in the incidence of untreated clinical malaria of 160 (103–221) cases per 1000 people in Guinea, 207 (139–296) cases per 1000 people in Sierra Leone, and 119 (81–172) cases per 1000 people in Liberia. Although Liberia has lower and less seasonal transmission (figure 1B), it had the highest treatment coverage before the present Ebola outbreak (55·7% receiving antimalarial treatment for fever compared with 28·1% in Guinea and 44·1% in Sierra Leone; figure 1C)^11,12,14^ and hence we estimated it to have had the highest proportional increase in untreated cases of the three countries. In all three countries, most untreated cases were in young children (48% [44–51] in children younger than 5 years, 38% [36–40] in children aged 5–15 years, and 14% (13–16) in people older than 15 years), resulting in little overlap with cases of Ebola virus disease (13·8% of which occur in children younger than 15 years).^2^

Most additional untreated cases caused by the Ebola epidemic would have occurred in the absence of Ebola virus disease but would have been treated because the high prevalence of malaria means that most transmission occurs among individuals who are sufficiently immune to be asymptomatic.^15,16^ However, a small proportion of the additional untreated cases are new cases (18% [13–24] in Guinea, 11% [7–15] in Liberia, and 14% [10–18] in Sierra Leone; figure 2), which are predicted to occur because of the effect of reduced malaria treatment on malaria transmission (figure 2).

We estimated that cessation of distribution of insecticide-treated bednets because of the Ebola epidemic would lead to a further 0·84 million (0·80 million to 1·01 million) cases of malaria in 2014, representing a 9·5% (8·7–12·8) increase. Moreover, this additional burden could accelerate sharply in 2015 if bednet use does not recover, with an estimated 2·7 million (1·9 million to 3·8 million) additional cases if coverage does not return to previous levels before the rainy season (appendix).

**Figure 3:**
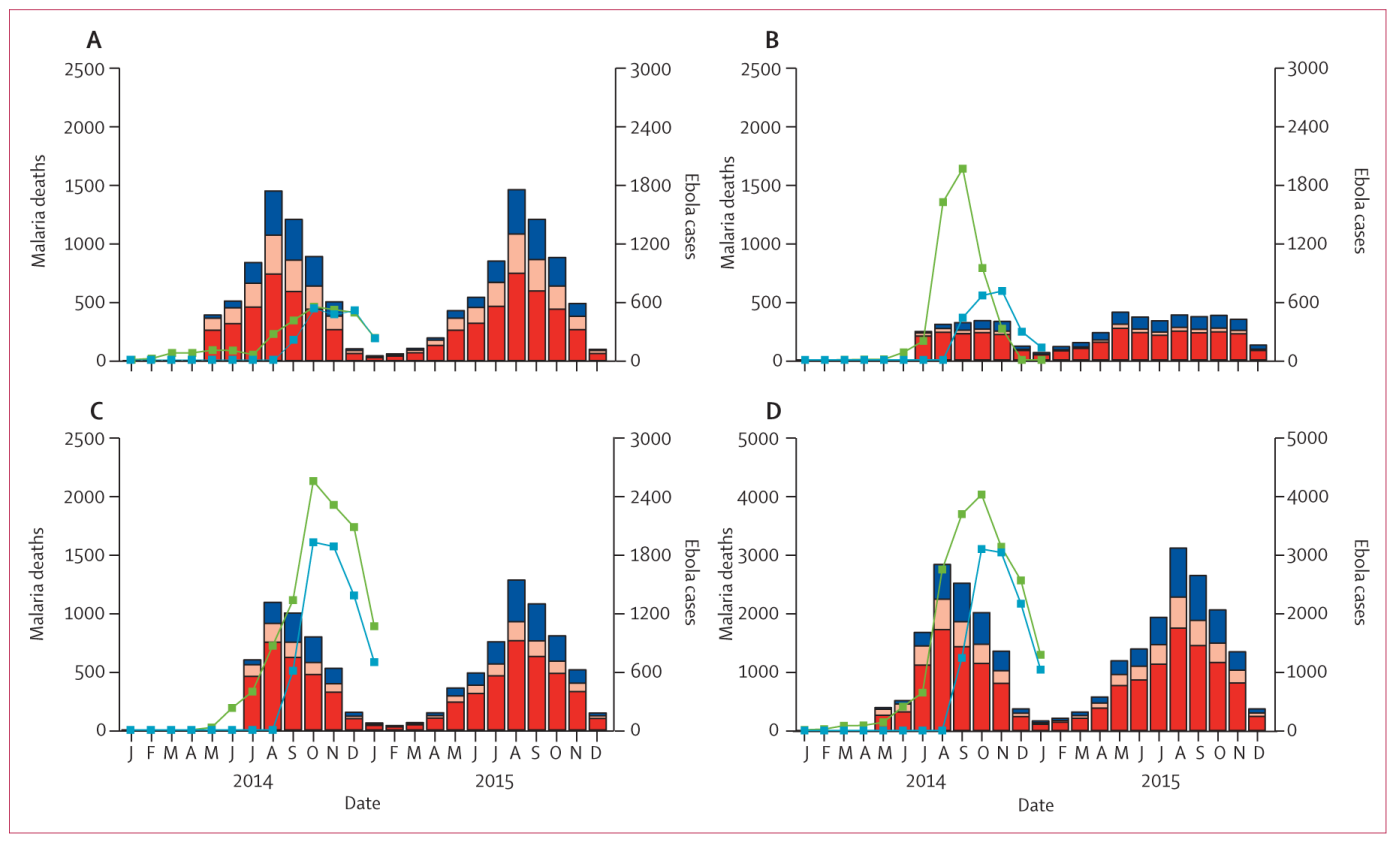
Effect of health-systems failure on malaria deaths For Guinea (A), Liberia (B), Sierra Leone (C), and the combined total (D). Red bars show additional deaths in individuals who would otherwise have been treated with an artemisinin-based combination therapy (ACT) and recovered, pink bars show additional deaths in individuals who would not have received ACT or failed to respond to ACT but would have otherwise recovered after hospital care, and blue bars show additional deaths caused by the additional malaria cases attributable to increased malaria transmission. Green lines show probable and confirmed Ebola cases from patient databases, blue lines show Ebola cases from WHO situation reports.

In the absence of clinic and hospital care, we estimated an increase in malaria-attributable mortality of 35% (25–49) in Guinea, 50% (34–71) in Sierra Leone, and 62% (40–94) in Liberia. This increase represents 10 900 additional malaria deaths (5700–21 400) over this period: 5600 (3000–11 100) in Guinea, 3900 (2000–7600) in Sierra Leone, and 1500 (700–2900) in Liberia (figure 1D). Most of these deaths are estimated to occur in people who would otherwise have recovered because of prompt access to first-line treatment with an effective antimalarial drug (figure 3). The small effect that disruption of first-line treatment has on transmission also means these numbers can be scaled to approximate any intermediate scenario where health-care provision has not been entirely disrupted (eg, a 75% reduction in health-care provision would result in 8000 [4200–15 700] additional malaria deaths, representing 73·4% [72·5–74·3] of the total in the absence of any provision; appendix). By contrast, disruption of delivery of insec ticide-treated bednets predominantly affects malaria transmission and hence additional disruption increases the number of cases overall. As a result, additional mortality caused by disruption of bednet delivery is less sensitive to how well the health system functions compared with before the Ebola epidemic, with the additional cases in our simulation leading to an additional 3900 deaths (2700–5500) in the absence of any health system or 3700 (2500–5100) additional deaths when the number of cases of uncomplicated malaria treated and hospital admissions for severe malaria has fallen by only 50%.

If usual health-care provision remains disrupted throughout 2015, we predict 46 400 (28 900–73 000) further deaths from malaria across the three countries compared with an estimated 31 800 deaths (20 500–47 800) if health systems return to function at the level before the Ebola epidemic (table).

**Figure 4:**
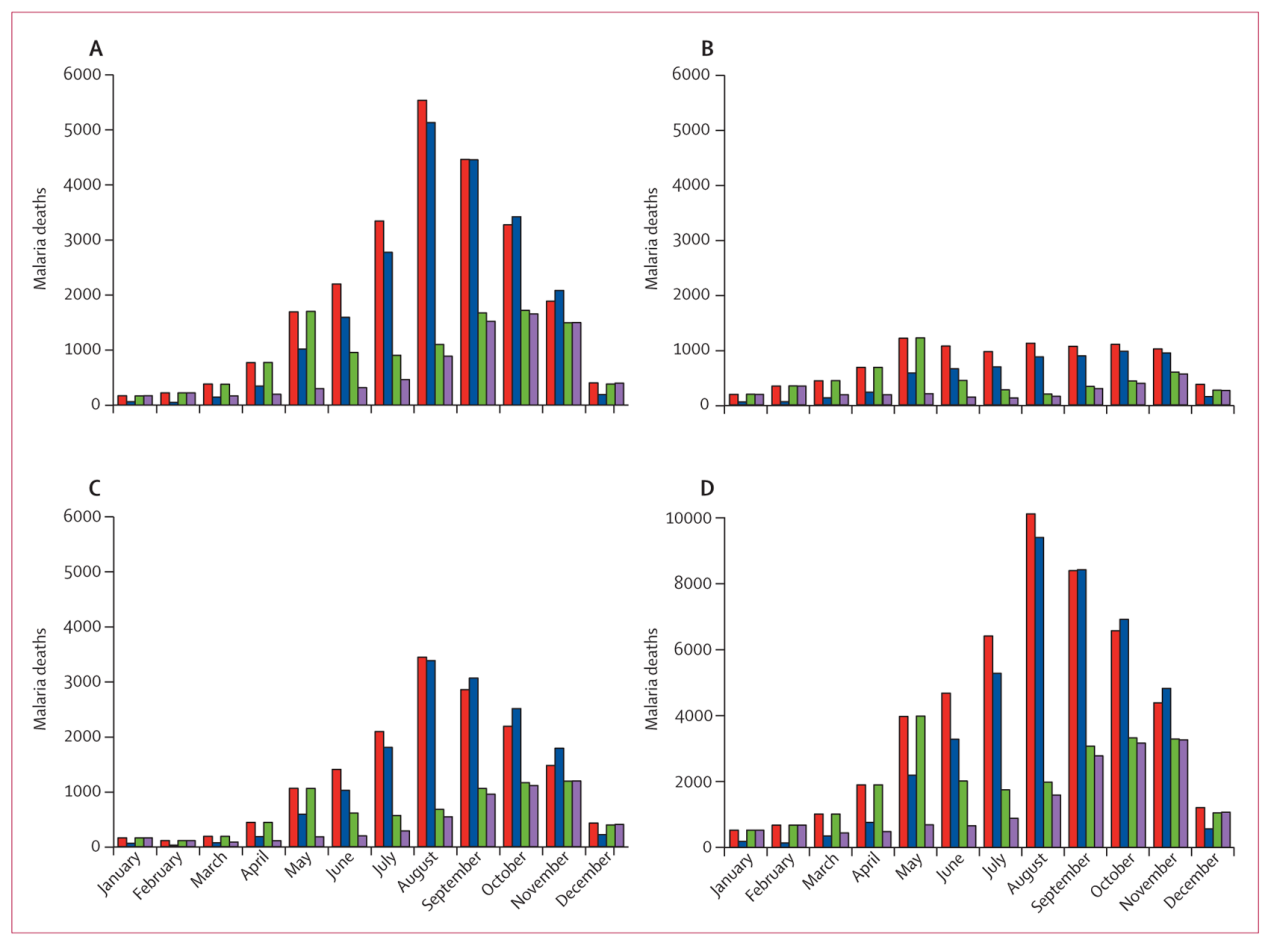
Estimated effect of mass drug administration on malaria deaths during 2015 For Guinea (A), Liberia (B), Sierra Leone (C), and the combined total (D). Red bars show predicted monthly malaria deaths in the absence of mass drug administration or any malaria treatment, blue bars show predicted monthly malaria deaths with mass drug administration with dihydroartemisinin–piperaquine for 3 months beginning in January 2015, the green bars show mass drug administration for 3 months beginning in June 2015, and the purple bars show mass drug administration for 6 months beginning in January 2015. All scenarios assume 70% coverage of the population and that the health system does not continue to function throughout the year.

Assuming that Ebola transmission will continue to decrease and health systems will begin to recover, our simulations suggest that a campaign of mass drug administration (assuming 70% coverage with arte-mether–piperaquine) in three rounds in consecutive months beginning in January, 2015, would reduce the burden of febrile malaria cases in the short term, leading to a 40% reduction in malaria cases and deaths in the first 6 months of 2015. However, because malaria transmission peaks at around July in these countries, our simulations support WHO’s guidance that, after two to three rounds, the need for continuing mass drug administration should be assessed (figure 4). If the health system remains compromised throughout 2015, we estimate that six rounds of mass drug administration would reduce the number of malaria attributable deaths to 21 200 (13 000–33 000) with artesunate–amodiaquine or to 14 000 (8700–20 200) with artemether–piperaquine at 70% coverage (table).

Disruption to delivery of insecticide-treated bednets in 2014 will probably have a large effect on both malaria cases and deaths in 2015, and hence providing bednets as early as possible in the year, either alongside mass drug administration or in a separate top-up campaign will probably greatly mitigate the effect of Ebola virus disease on malaria (appendix). In our simulation, a campaign to replace all insecticide-treated bednets and return coverage to pre-Ebola levels in February, 2015, reduced the number of additional deaths attributable to disruption of bednet distribution by 65% (64–66).

## Discussion

In a scenario in which no patients with malaria receive first-line treatment or hospital care, we estimate that the Ebola epidemic could have resulted in many additional deaths in 2014 in Guinea, Liberia, and Sierra Leone. Because of the low proportion of deaths attributable to new cases of infection, this worst-case scenario provides a readily adjustable benchmark with which to assess the probable effect of health-system disruption if the real-life effect on treatment and hospital admission can be assessed more accurately in the aftermath of the crisis.

Our estimate does not incorporate the additional effect of disruption to delivery of bednets, which we showed could result in an increase in malaria incidence, emphasising the importance of ensuring the bednets are in place for the forthcoming transmission season. It also excludes the effect of Ebola virus disease on maternal malaria morbidity. Malaria has been estimated to cause 430 000 infections during pregnancy and 31 100 low birthweight deliveries if adequate health care is not available in these three countries based on 2010 levels of transmission.^17^ Provision of intermittent preventive treatment for pregnant women is high in Sierra Leone (61·7% of pregnant women) and Liberia (47·6%),^12,14^ although lower in Guinea (17·8%);^11^ therefore, Ebola virus disease preventing access to health care will probably have a substantial effect on malaria morbidity in pregnant women and babies. As a result, the overall indirect effect of Ebola virus disease on malaria will probably be equal or greater than the direct effect of the virus itself.

By including the additional effect of Ebola virus disease on health care for other common diseases in the affected areas, as well as the effect that a non-functioning health system has on broader maternal and infant health, these estimates provide an indication of the true size of the humanitarian crisis caused by the outbreak of Ebola virus disease (panel). They also suggest that these effects will probably continue well after the epidemic has been contained while both the health system itself, and trust in it, recover.

Panel **Research in context**

**Systematic review**

We searched PubMed with the terms “malaria” and “Ebola”, to identify estimates of the effect of the ongoing Ebola epidemic on malaria burden, published up to Feb 1, 2015. We found no relevant studies and one news report,^4^ which did not attempt to estimate the effect of the Ebola epidemic on malaria.

**Interpretation**

We estimate that the indirect effect of the Ebola epidemic on malaria morbidity and mortality is probably of similar size as the public health burden directly caused by Ebola virus disease. This finding suggests that measures to prevent malaria infection, such as the emergency mass drug administration suggested by WHO, are urgently needed while health systems recover.

Our results also show the potential effect of mass drug administration, as recently recommended in WHO guidance for malaria control in affected areas. Such strategies are logistically challenging and, particularly in an Ebola epidemic, need careful and thorough social mobilisation and community engagement. However, we estimate that mass drug administration could substantially reduce additional malaria burden. An additional benefit of preventive interventions is the corresponding reduction in the need for treatment and hence a reduction in the burden imposed on already strained health systems. One of the key factors determining the success of such a campaign will be the level of coverage achieved. We assumed 70% coverage, but our results show that lower coverage could still have a substantial effect. Our results also provide some guidance for the timing of the campaigns. Immediate implementation of these strategies, as is occurring in affected areas in Sierra Leone,^5^ is likely to help with the pressing short-term task of rapidly identifying Ebola cases by alleviating the burden of malaria on the health system.

However, in Sierra Leone and Guinea, malaria transmission is highly seasonal, with cases expected to have fallen around November and December, 2014. Malaria cases will begin to rise again at the start of the next rainy season (May and June, 2015) and hence could provide an additional challenge to ending the Ebola epidemic. Thus, the feasibility of extending mass drug administration campaigns beyond three rounds should be carefully considered.

## Supplementary Material

Supp1

## Figures and Tables

**Table:** Predicted malaria cases and deaths in 2015, by country

	Guinea		Liberia		Sierra Leone		Total	
	Cases, millions	Deaths	Cases, millions	Deaths	Cases, millions	Deaths	Cases, millions	Deaths
Baseline	5·3 (3·8–7·2)	17 200 (10 300–28 400)	1·6 (1·1–2·2)	4300 (2700–6900)	3·4 (2·3–4·6)	9900 (5900–16 400)	10·3 (8·1–12·7)	31 800 (20 500–47 800)

Additional	0·3 (0·2–0·6)	6200 (3100–11 700)	0·2 (0·1–0·4)	3000 (1600–6000)	0·3 (0·1–0·6)	5300 (2600–9900)	0·9 (0·5–1·3)	14 800 (8300–24 800)

Total	5·6 (3·9–7·8)	23 600 (13 700–39 800)	1·8 (1·2–2·5)	7300 (4400–12 700)	3·7 (2·5–5·1)	15 200 (8700–26 100)	11·5 (8·7–14·0)	46 400 (28 900–73 000)

3-month MDA from January								
Dihydroartemisinin– piperaquine, 30% coverage	5·3 (3·7–6·8)	22 800 (13 400–34 100)	1·7 (1·0–2·2)	7000 (3800–10 800)	3·5 (2·3–4·5)	14 400 (9100–22 100)	10·5 (7·4–13·1)	44 100 (26 400–67 000)
Dihydroartemisinin– piperaquine, 50% coverage	5·0 (3·3–6·2)	21 200 (12 400–30 700)	1·5 (1·0–2·0)	6600 (3800–10 700)	3·3 (2·1–4·3)	13 500 (7400–20 200)	9·9 (6·5–12·2)	41 200 (23 700–61 500)
Dihydroartemisinin– piperaquine, 70% coverage	4·4 (2·9–5·8)	19 200 (11 600–28 100)	1·3 (0·9–1·8)	5500 (3200–8200)	3·0 (1·9–4·0)	12 800 (7000–19 500)	8·7 (5·7–11·4)	37 400 (22 000–55 600)
Artesunate–amodiaquine, 70% coverage	4·5 (3·2–6·3)	20 000 (12 100–28 400)	1·4 (0·9–1·8)	6000 (3300–8800)	3·1 (2·0–4·1)	13 300 (7200–19 700)	8·9 (6·2–12·1)	39 400 (22 700–56 500)

3-month MDA from July								
Dihydroartemisinin– piperaquine, 30% coverage	4·4 (3·1–5·5)	18 600 (10 800–28 800)	1·6 (1·0–2·1)	6500 (3500–9500)	3·1 (2·0–4·0)	12 700 (6900–21 100)	9·2 (6·1–11·7)	37 800 (21 300–58 500)
Dihydroartemisinin– piperaquine, 50% coverage	3·5 (2·3–4·4)	15 000 (9200–21 600)	1·4 (0·9–1·8)	5900 (3200–8600)	2·7 (1·7–3·5)	11 600 (6700–16 400)	7·7 (5·0–9·6)	32 400 (19 200–46 000)
Dihydroartemisinin– piperaquine, 70% coverage	2·7 (1·9–3·4)	11 400 (6700–17 900)	1·2 (0·8–1·5)	4900 (2700–7700)	2·2 (1·5–2·9)	9700 (5800–13 800)	6·0 (4·2–7·9)	26 100 (15 500–38 300)
Artesunate–amodiaquine, 70% coverage	3·7 (2·5–5·2)	16 400 (10 300–24 500)	1·4 (0·9–1·8)	6100 (3600–9300)	2·9 (1·8–3·8)	12 100 (6500–17 900)	7·9 (5·2–10·6)	34 500 (20 600–50 200)

6-month MDA from April								
Dihydroartemisinin– piperaquine, 30% coverage	3·9 (2·7–5·0)	16 400 (10 100–23 600)	1·2 (0·8–1·6)	5200 (3200–7400)	2·5 (1·7–3·5)	7300 (4400–10 400)	7·6 (5·3–10·8)	28 800 (17 900–40 700)
Dihydroartemisinin– piperaquine, 50% coverage	2·8 (1·9–3·7)	12 300 (7500–17 400)	0·9 (0·6–1·2)	3700 (2000–5800)	1·8 (1·3–2·4)	5300 (2800–8300)	5·5 (3·8–7·1)	21 300 (12 700–30 200)
Dihydroartemisinin– piperaquine, 70% coverage	1·8 (1·3–2·4)	7900 (4900–10 900)	0·6 (0·4–0·8)	2500 (1400–4000)	1·3 (0·8–1·6)	3600 (2100–5900)	3·8 (2·8–4·9)	14 000 (8700–20 200)
Artesunate–amodiaquine, 70% coverage	3·0 (1·9–3·9)	12 300 (7800–18 500)	0·9 (0·5–1·2)	3700 (2000–6000)	1·9 (1·3–2·5)	5400 (2900–8800)	5·7 (3·7–7·4)	21 200 (13 000–33 000)

Data are n (95% credible interval). Baseline shows the estimates if health systems return to the level of functioning from before the Ebola epidemic. The additional cases are those that are predicted to occur if the health systems continue not to function throughout 2015 and no other remedial action is taken. The mass drug administration (MDA) scenarios show the total cases that are predicted without a return to a functioning health system.
