# Supplementary material for: Malaria morbidity and mortality in Ebola-affected countries caused by decreased health-care capacity, and the potential effect of mitigation strategies: a modelling analysis
Source: Lancet Infect Dis. Author manuscript; Available in PMC 2016 Apr 7. (PMC4824180; doi:10.1016/S1473-3099(15)70124-6)
Supplement: Supp1 [file NIHMS773812-supplement-Supp1.pdf]

THE LANCET Infectious Diseases

## Supplementary webappendix

This webappendix formed part of the original submission and has been peer reviewed. We post it as supplied by the authors.

Supplement to: Walker PGT, White MT, Griffin JT, Reynolds A, Ferguson NM, Ghani AC. Malaria morbidity and mortality in Ebola-affected countries caused by decreased health-care capacity, and the potential effect of mitigation strategies: a modelling analysis. *Lancet Infect Dis* 2015; published online April 24. http://dx.doi.org/10.1016/S1473-3099(15)70124-6.

## Impact of Ebola upon malaria in affected countries and potential mitigation strategies.

Patrick GT Walker*, Michael T White, Jamie T Griffin, Alison Reynolds, Neil M Ferguson, Azra C Ghani

*MRC Centre for Outbreak Analysis & Modelling, Imperial College London*

### Supporting Information

This file includes supplementary results referred to within the main paper and a short summary of the technical details of the model. The model has been developed over a number of years with the combined intervention model for *P. falciparum* malaria transmission first presented in detail in Griffin et al. 2010[1]. The impact of mass drug administration strategies using this model and validation of the model parameters compared to past studies of MDA was published in Okell et al. 2011[2].

The parameters underlying the transmission model in the absence of interventions were updated in 2014 to fit to a wider range of field studies on the relationship between parasite prevalence and clinical disease[3]. This resulted in a similar all-age relationship to that identified empirically and used in the burden estimation methods[4] which have subsequently been adopted by WHO to produce country-level estimates of malaria cases for the annual World Malaria Report[5].

The vector model was further developed by White et al. (2011) to capture seasonal variation in mosquito density and the impact that vector interventions have on reproduction by capturing the larval stages of the mosquito lifecycle[6]. This model is included here and summarised below.

In parallel, we further developed the way that the model captures the effect of treatment for case management and through mass drug administration by incorporating distributions that capture the pharmacokinetics and pharmacodynamics for two artemisinin-combination therapies (Artemether-Lumefantrine (AL) and Dihydroatermisinin-Piperaquine (DHA-P))[7].

These updates have all been combined into the single model used here. Section (1) contains supplementary tables and figures referred to in the main text. Section (2) summarises the mathematical details and parameters for the baseline transmission model as presented in Griffin et al [3]. Section (3) outlines the decision-tree used to extend the case incidence outputs to estimate mortality and the parameters and sensitivity ranges used. Section (4) outlines the site-specific parameters used to estimate transmission levels in 2014 including treatment rates and LLIN coverage prior to the Ebola epidemic. Section (5) summarises the implementation of MDA strategies in the model and the parameters used for the two drugs considered in the main text. Finally, in Section (6) we provide a comparison of our estimated baseline burden in 2015 (cases and deaths) with those estimated by others.

## 1. Supplementary Results

**Table S1: Sensitivity analysis of the number of deaths attributable to Ebola according to different assumed reductions in healthcare capacity.**

| % Reduction in healthcare capacity | Guinea | Liberia | Sierra Leone | Total |
|---|---|---|---|---|
| **Estimated total (thousands) malaria deaths attributable to Ebola in 2014 (95% CrIs)** | | | | |
| 25% reduction | 1.3 (0.7-2.5) | 0.3 (0.2-0.7) | 0.9 (0.5-1.7) | 2.5 (1.3-4.9) |
| 50% reduction | 2.7 (1.4-5.2) | 0.7 (0.4-1.4) | 1.8 (0.9-3.6) | 5.2 (2.7-10.2) |
| 75% reduction | 4.1 (2.2-8.1) | 1.1 (0.5-2.1) | 2.8 (1.5-5.5) | 8.0 (4.2-15.7) |
| 100% reduction | 5.6 (2.9-11.1) | 1.5 (0.7-2.9) | 3.9 (2.0-7.6) | 10.9 (5.7-21.4) |

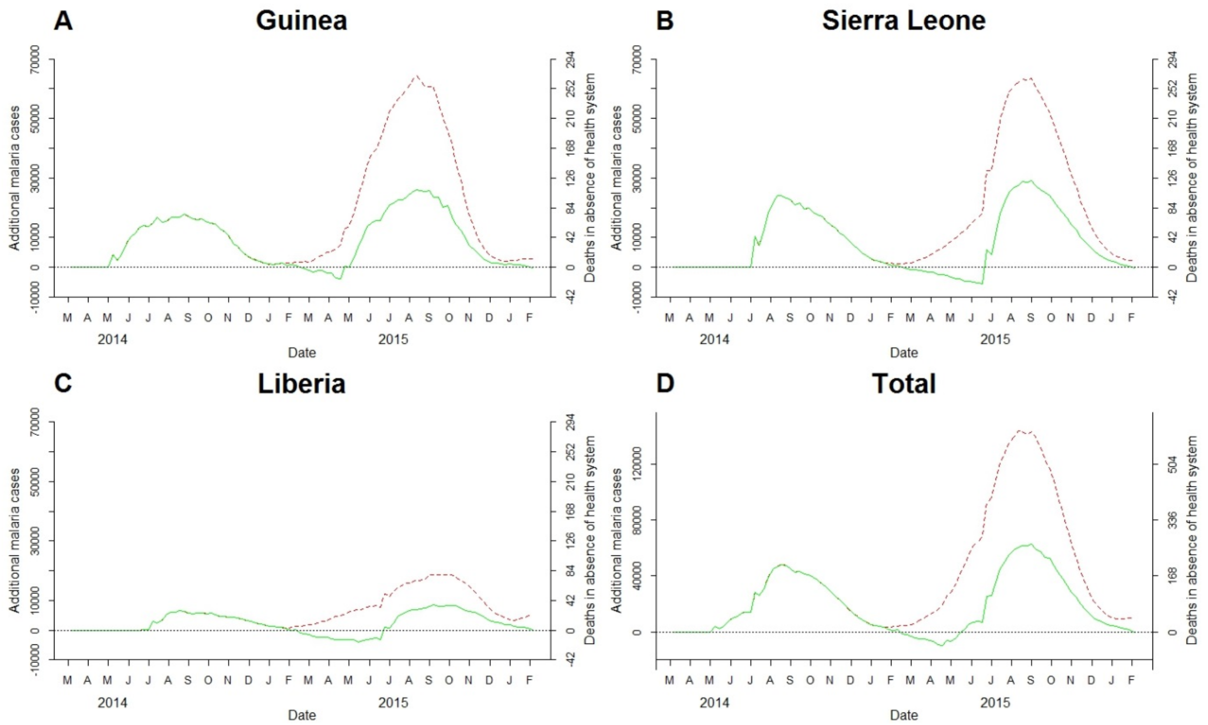


**Figure S1| Potential Impact of disruption to ITN delivery** Figure shows the potential effect upon weekly case incidence if ITN renewal stops following the second month of sustained transmission of Ebola. In each plot the dashed red lines show the number of additional cases relative to those that would have occurred with constant ITN usage in the event that ITNs are never renewed. The green lines show the same numbers but where ITN delivery at pre-Ebola levels of coverage is delayed until the first quarter of 2015 (green lines dip below zero soon after these nets are delivered as the efficacy of nets delivered earlier in the year in the absence of Ebola would have waned due to attrition and wear and tear). The right-hand axis shows number of deaths in the absence of treatment of uncomplicated malaria or hospitalisation of those with severe disease.

## 2. Baseline transmission model

We describe the model below in its deterministic compartmental framework. However, for the analysis, an equivalent stochastic individual-based version was implemented as it is more efficient for multiple interventions. The only modification to this structure in the individual-based version is the non-exponential distributions of treatment and prophylaxis as outlined in Section 3.

Susceptible individuals (S) become infected at a rate $\Lambda$ which is determined by the time-varying entomological inoculation rate (EIR) in the mosquito population. Following a delayed latent period of approximately 12 days, infected individuals either develop symptomatic clinical disease (with probability $\phi$) or move into the asymptomatic infection state (A), where the probability of developing symptomatic disease is a function of exposure-drive immunity (see below). Those that develop disease are either effectively treated (T) or untreated (D). Treated individuals experience a period of prophylaxis (P) depending on the drug used and recover to the susceptible compartment, whilst untreated individuals eventually recover naturally from symptoms and move to the asymptomatic state (A). Since deaths are only a small proportion of the total cases (see section 2) we do not explicitly model deaths in the transmission component of the model. Asymptomatic individuals move to sub-patent infection (U) during their course of infection to capture residual onward transmission that occurs from individuals with low density parasitaemia. Super infection can occur from all infected states. All states are further stratified by the level of exposure to mosquitoes (not shown explicitly here). We do not model disease-induced mortality and assume a constant non-growing population with a fixed death rate to simulate an exponential distribution with mean of 21 years parameterised with data from Tanzania[8]. Hence the birth rate of the population (with all births into the susceptible population at age 0) is set to equal the death rate – for simplicity we do not include these terms in the equations below.

The partial differential equations for the human dynamics are given by:

$$\begin{aligned}
\frac{\partial S}{\partial t}+\frac{\partial S}{\partial a}+\frac{\partial S}{\partial \zeta} &= -\Lambda S + P/d_P + U/d_U \\
\frac{\partial T}{\partial t}+\frac{\partial T}{\partial a}+\frac{\partial T}{\partial \zeta} &= \phi f_T \Lambda (S+A+U) - T/d_T \\
\frac{\partial D}{\partial t}+\frac{\partial D}{\partial a}+\frac{\partial D}{\partial \zeta} &= \phi (1-f_T) \Lambda (S+A+U) - D/d_D \\
\frac{\partial A}{\partial t}+\frac{\partial A}{\partial a}+\frac{\partial A}{\partial \zeta} &= (1-\phi)\Lambda (S+U) + D/d_D - \phi \Lambda A - A/d_A \\
\frac{\partial U}{\partial t}+\frac{\partial U}{\partial a}+\frac{\partial U}{\partial \zeta} &= A/d_A - U/d_U - \Lambda U \\
\frac{\partial P}{\partial t}+\frac{\partial P}{\partial a}+\frac{\partial P}{\partial \zeta} &= T/d_T - P/d_P
\end{aligned} \tag{1}$$

where $t$ denotes time, $a$ denotes age and $\zeta$ denotes the risk group (relative biting rate). Here $\Lambda$ is the force of infection, $\phi$ is the probability of developing symptomatic clinical

disease following infection, $f_T$ is the proportion of clinical malaria cases that are effectively treated (see Section 3) and $d_D$, $d_T$, $d_P$, $d_A$ and $d_U$ denote the mean duration of their respective states.

Variation in exposure to mosquitoes is included to capture both geographic/risk heterogeneity and differences in body size. In the individual-based version model, each individual has a relative biting rate $\zeta$ which is drawn from a Log-Normal distribution with parameters $-\sigma^2/2$ and $\sigma$, parameterised such that $\zeta$ has a mean of 1 (see for further details).

Letting $EIR_0$ denote the mean EIR in adults, the EIR ( $EIR$ ) and force of infection ( $\Lambda$ ) at age $a$ are given by:

$$\begin{aligned} EIR &= EIR_0\zeta\left(1-\rho\exp(-a/a_0)\right) \\ \Lambda &= EIRb \end{aligned} \qquad (2)$$

where $b$ is the probability of infection following the bite of an infectious mosquito, and $\rho$ and $a_0$ are parameters to capture the variation with age as a proxy for body size.

The duration of disease episodes is fixed at 5 days to capture the high onward infectivity to mosquitoes that occurs for this time period in both treated and untreated infections. Those who are effectively treated are then assumed to no longer be infectious whilst untreated infections enter the asymptomatic state. Recurrent bouts of disease can occur from this state through superinfection but we do not include recurrent bouts from existing malaria infections.

Immunity is incorporated in the model at different stages: a low level of pre-erythrocytic immunity that is acquired with exposure and reduces the probability of infection, *b*; maternal immunity that protects against disease in the first year of life; naturally-acquired immunity to blood-stage infection that increases with exposure and reduces the probability of developing clinical disease; and a level of blood-stage immunity that develops more slowly but reduces the detectability of asymptomatic infections as well as their onward infectiousness to mosquitoes. Full details of the model and associated parameters for immunity are given in Griffin et al. 2014[3].

A summary of the human parameters for the baseline transmission and their 95% credible intervals where fitted is given in Table S2.

**Table S2: Parameters for the baseline transmission model: human component. See [3] for sources for prior distributions.**

| Parameter | Symbol | Estimate (95% credible interval) |
|---|---|---|
| **Human infection duration (days)** | | |
| Latent period | $d_E$ | 12 (fixed) |
| Patent infection | $d_I$ | 200 (fixed) |
| Clinical disease (treated) | $d_T$ | 5 (fixed) |
| Clinical disease (untreated) | $d_D$ | 5 (fixed) |
| Sub-patent infection | $d_U$ | 110 (87,131) |
| Prophylaxis following treatment | $d_P$ | Based on drug PK/PD profile[3] |
| **Infectiousness to mosquitoes** | | |
| Lag from parasites to infectious gametocytes | $t_l$ | 12.5 days (fixed) |
| Untreated disease | $c_D$ | 0.068 day$^{-1}$ (0.039, 0.122) |
| Treated disease | $c_T$ | Drug-dependent[7] |
| Sub-patent infection | $c_U$ | 0.0062 day$^{-1}$ (0.00056, 0.018) |
| Relative infectiousness of asymptomatic state based on the probability of detection | $\gamma_I$ | 1.82 (0.603, 8.54) |
| **Age and heterogeneity** | | |
| Age-dependent biting parameter | $\rho$ | 0.85 (fixed) |
| Age-dependent biting parameter | $a_0$ | 8 years (fixed) |
| Variance of the log heterogeneity in biting rates | $\sigma^2$ | 1.67 (fixed) |
| **Parameters depending on immunity** | | |
| Probability that a human infection leads to disease | $\phi$ | See Griffin et al. (2014) for further details[3] |
| Probability of human infection following the bite of an infectious mosquito | $b$ | |

The vector component of the model is captured in a deterministic compartmental formulation capturing the full lifecycle of the mosquito. Female adult mosquitoes lay $\beta$ eggs per day (E) which then develop through the larval stages (captured here as “early” $L_1$ for instars 1 and 2 and “late” $L_3$ for instars 3 and 4) through to the pupal stage Pu. All three stages are subject to density-dependent mortality. Surviving pupae then emerge as adult mosquitoes (M) and we track only the adult female mosquitoes (assumed to be 50% of the emerging adult mosquitoes). The set of differential equations for the mosquito lifecycle are:

$$\frac{dL_1}{dt} = \beta M - \mu_{L_1}\left(1+\frac{L_1+L_3}{K}\right)L_1 - \frac{L_1}{d_{EL}}$$
$$\frac{dL_3}{dt} = \frac{L_1}{d_{EL}} - \mu_{L_3}\left(1+\gamma\frac{L_1+L_3}{K}\right)L_3 - \frac{L_3}{d_L}$$
$$\frac{dPu}{dt} = \frac{L_3}{d_L} - \mu_P Pu - \frac{Pu}{d_P} \quad (3)$$
$$\frac{dS_M}{dt} = \frac{Pu}{2d_P} - \mu S_M$$

where $\beta$ is the number of surviving female eggs oviposited per adult female mosquito per unit time, $\mu_{L_1}$ is the death rate of early stage larvae at low larval density, $\mu_{L_3}$ is the death rate of late-stage larvae at low larval density, $\mu_P$ is the death rate of pupae, $\mu^v$ is the death rate of adult mosquitoes, $d_{EL}$ is the mean duration of the early larval stage, $d_L$ is the mean duration of the late larval stage, $d_P$ is the mean duration of the pupal stage, $K(t)$ is the time-varying carrying capacity, and $\gamma$ is the additional contribution of late-stage larvae to the carrying capacity as compared to early-stage larvae (to account for their larger size).

Emerging adult mosquitoes are assumed to be susceptible to infection ($S_M$) and become infected at a rate dependent on the infectiousness of the human population. The force of infection acting on mosquitoes is the sum of the contribution to mosquito infection from the different human infectious states stratified by age (*a*) and heterogeneity in exposure ($\zeta$)

$$\Lambda_M(t) = \frac{Q_0}{\omega\delta}\int_\zeta\int_a \zeta\psi(a)\big(c_D D(\zeta,a,t-t_l) + c_T T(\zeta,a,t-t_l) + c_A A(\zeta,a,t-t_l) + c_U U(\zeta,a,t-t_l)\big)\,da\,d\zeta \quad (4)$$

where $c_D$, $c_T$, $c_A$ and $c_U$ are the onward infectivity to mosquitoes of these different states, estimated from fitting to infectivity studies from four sites (Details of these fits are given in the publication by Griffin et al. (2014) [3]). Here $t_l$ is the time-lag between parasitaemia with asexual parasite stages and gametocytaemia (infectivity to mosquitoes) to account for the lag in gametocyte development characteristic of *P. falciparum*. The integral over age $a$ is across the full population (0 to 100 years). $\omega$ is a normalizing constant for the biting rate over ages given by the expression:

$$\omega = \int_0^\infty \psi(a)g(a)\,da \quad (5)$$

where *g(a)* is the human age distribution (assumed here to be at equilibrium) and $\psi(a)$ is the distribution of the biting rate by age which is included to account for differences in body sizes. The biting rate on humans is given by $\frac{Q_0}{\delta}$ where $\delta$ is the mean time between feeds and $Q_0$ is the proportion of bites that are taken on humans (the degree of anthropagy of the vector). $c_D$, $c_T$, $c_A$ and $c_U$ are independent model parameters and the infectiousness of the

asymptomatic stage $c_A$ is intermediate to that of the clinical disease and sub-patent states - $c_U + (c_D - c_U)q^{\gamma_I}$ - where q and $\gamma_I$ are parameters in the immunity model[3].

Once infected ($E_M$), the adult mosquitoes experience a period of sporogony after which they become infectious to humans ($I_M$). Given the short lifespan of mosquitoes, they are assumed to remain infectious until they die. The dynamics of infection in the mosquito is therefore given by the set of differential equations,

$$\frac{dS_M}{dt} = \frac{Pu}{2d_P} - \Lambda_M S_M - \mu S_M$$
$$\frac{dE_M}{dt} = \Lambda_M S_M - \Lambda_M (t - \tau_M) S_M (t - \tau_M) P_M - \mu E_M \quad (6)$$
$$\frac{dI_M}{dt} = \Lambda_M (t - \tau_M) S_M (t - \tau_M) P_M - \mu I_M$$

where $P_M = \exp(-\mu \tau_M)$ is the probability that a mosquito survives the period from acquiring infection until sporozoites appear in the salivary glands ($\tau_M$, extrinsic incubation period or EIP), $\mu$ is the death rate and $\Lambda_M$ is the force of infection acting on adult mosquitoes.

The resulting exposure to humans is then defined by the mean EIR in adults, $EIR_0$, which depends on the proportion of infected mosquitoes, $I_M$:

$$EIR_0 = I_M \alpha / \omega \quad (7)$$

A summary of the vector parameters for the baseline transmission model and their 95% credible intervals where fitted is given in Table S3. We assumed larval capacity K(t) is directly proportional to the smoothed estimated daily rainfall reconstructed from the first three frequencies of the Fourier-transformed data (see later) . Thus

$$K(t) = K^* \frac{R(t)}{E[R(t)]} \quad (8)$$

where R(t) is the daily rainfall and K* is the larval capacity at mean rainfall. K* was scaled to give the baseline level of endemicity in each second administrative unit (see later).

**Table S3: Parameters for the baseline transmission model: vector component. See[6] for sources of prior information for the parameters.**

| Parameter | Symbol | Estimate used |
|---|---|---|
| Daily mortality of adults (based on *An.gambiae* complex) | $\mu$ | 0.132 day$^{-1}$ (fixed) |
| Per capita daily mortality rate of early instars (low density) | $\mu_E^0$ | 0.034 (0.024-0.044) day$^{-1}$ |
| Per capita daily mortality rate of late instars (low density) | $\mu_L^0$ | 0.035 (0.025-0.044) day$^{-1}$ |
| Per capita daily mortality rate of pupae | $\mu_P$ | 0.25 (0.18-0.32) day$^{-1}$ |
| Duration of gonotrophic  cycle | $\delta$ | 3 days |
| Development time of early larval instars | $d_{EL}$ | 6.64 (4.82-8.53) days |
| Development time of late larval instars | $d_L$ | 3.72 (2.03-5.61) days |
| Development time of pupae | $d_P$ | 0.64 (0.07-1.47) days |
| Maximum eggs laid per oviposition | $e_{ov}^m$ | 93.6 |
| Number of eggs laid per day per mosquito | $\beta$ | 21.19 (11.57-25.31) day$^{-1}$ |
| Relative effect of density dependence on late instars relative to early instars | $\gamma$ | 13.25 (9.82-17.51) |
| Environmental carrying capacity | $K$ | See expression above |
| Extrinsic incubation period | $\tau_M$ | 10 days |
| Force of infection on adult mosquitoes | $\Lambda_M$ | See expression above. |

We also included bed net usage in this model by introducing modifications to the probability that a mosquito is repelled from the house, the probability that it is killed on contact with a bed net and the probability that it bites successfully. Full details and parameters for this model are given in White et al. (2011)[6].

## 3. Extension of the model to estimate mortality

There is a paucity of data on malaria disease and mortality due to the lack of robust health reporting systems in countries with the highest burden. For this reason, the WHO currently rely on model-based estimates in 40 out of the 97 malaria-endemic countries[9]. Methods for improving malaria burden estimation were recently considered by an Evidence Review sub-group of the Malaria Policy Advisory Committee and resulted in several recommendations for improving the evidence base[9,10]. However, it should be borne in mind that, whilst we have attempted to obtain a sensible overall estimate as outlined below and capture a degree of the associated uncertainty, further work remains to obtain more robust estimates of the true burden of disease more generally.

The models described in Section 1 are used to predict the incidence of treated and untreated clinical disease by age and time. This is multiplied by the age-stratified population size to

obtain and estimate the number of clinical cases by age and over time. We then used a decision tree model to estimate the risk of mortality arising from a clinical case. This is illustrated in Figure S2. A proportion $f_T$ of cases are assumed to receive treatment. This is matched to district-level estimates of the proportion of children under 5 receiving antimalarial medication from the appropriate DHS/MIS survey (Table S4). Rather than completely curing all infections, this is assumed to reduce the probability of developing severe disease by a proportion $\eta$, reflecting both delays between onset and treatment due to variation in care-seeking behaviour and timely access to health services that are thought to be major determinants of the onset of severe disease[11–13], as well as variation in the risk of rapidly progressing to severe malaria with age and prior exposure[14]. A proportion of those developing severe disease (either untreated or not prevented by first-line treatment) will seek further care and be hospitalised, where we assume that they receive the current standard of care in these countries (intravenous quinine). Mortality in hospital is substantially lower than for cases that are not hospitalised, with our central estimate of 10% based on a recent review of hospital outcomes in a range of African care settings[15].

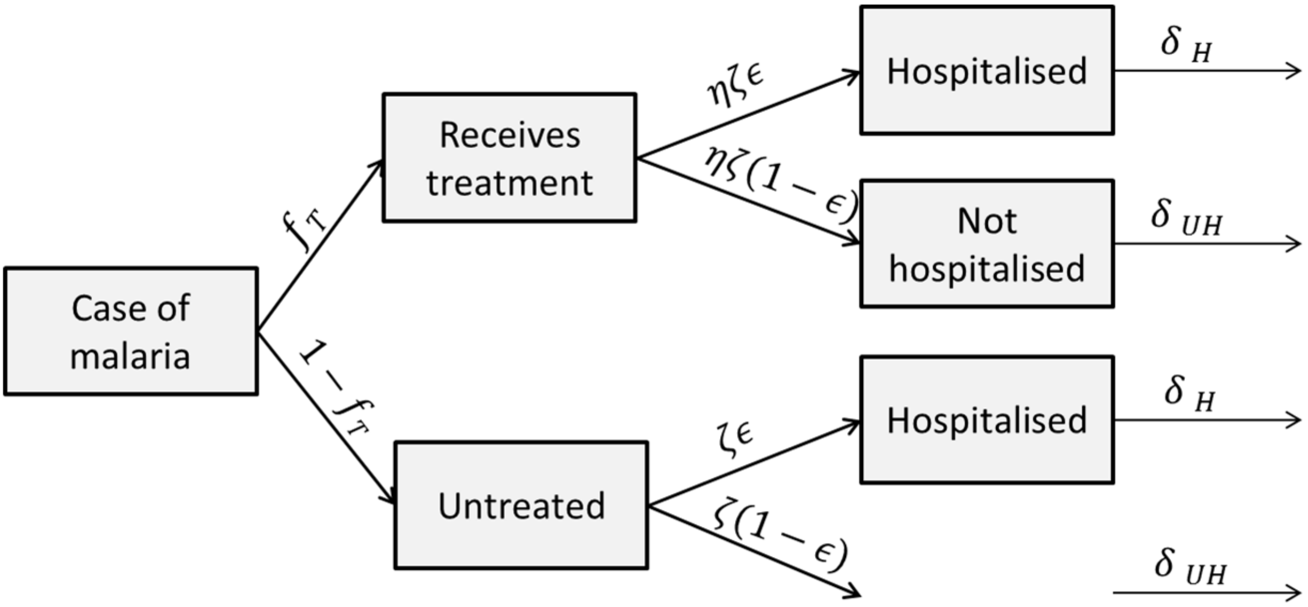


**Figure S2: Decision-tree for health system management of malaria cases** Figure shows model in the presence of a functioning health system. Arrows indicate proportions of individuals who continue onto the next stage. When the health system is not working individuals experience a case-fatality ratio of $\zeta\delta_{UH}$ as they go untreated and do not reach hospital. Only pathways involving both severe disease and death are shown, pathways involving recovery are excluded in the diagram (but included implicitly in the model) as they do not contribute to the number of deaths.

A number of studies have estimated case fatality ratios for malaria in the range 0.3% to >1%. Here we use the value currently applied for burden estimation in the World Malaria report (0.3%) as a central estimate[16] and capture this uncertainty in sensitivity bounds. As the proportion of untreated cases that develop severe disease is largely unknown we allow this to be determined by the other parameters and their assumed uncertainty. This is done by drawing the other model parameters from their specified prior distribution (see Table S4) and then back-calculating the severe disease probability α to achieve the case fatality drawn

from its prior distribution for the median country-level treatment rate of the three countries (44.1% in Sierra Leone, relative to 55.7% in Liberia and 28.1% in Guinea)[17–19]. This provides a range of 0.6-1%, a number which is a conservative estimate relative to previous analyses [20,21].

**Table S4: Parameters for the health system management model**

| Parameter | Symbol | Central estimate (range) prior to Ebola epidemic | Reference | Assumed value during Ebola epidemic |
|---|---|---|---|---|
| % of cases treated | $f_T$ | Setting specific | 17–19 | 0 |
| % of untreated cases developing severe disease | $\zeta$ | 0.7% (0.4%-1%) | Calculated from CFR | Same |
| Reduction in % developing severe disease due to treatment | $\eta$ | 50% (Triangular distribution between 25% and 75%) | See text | N/A |
| % of severe disease hospitalised | $\varepsilon$ | 10% (Triangular distribution between 5% and 20%) | Assumed | 0 |
| % mortality from severe disease in hospital | $\delta_H$ | 10% (Triangular distribution between 5% and 20%) | 15 | N/A |
| % mortality from severe disease outside hospital | $\delta_{UH}$ | 60% (Triangular distribution between 45% and 80%) | 20 | Same |
| CFR (based on Sierra Leone treatment level) | - | 0.3% (Triangular distribution between 0.2% and 0.4%) | 16 | Re-estimated from above parameters |

Our baseline scenario was that the health system had entirely failed (i.e $\varphi = 0$ and $\varepsilon = 0$ ) following two months of sustained Ebola transmission. We also carried out a sensitivity analysis looking at additional malaria cases and mortality when these parameters were reduced by 75%, 50% and 25% of their pre-Ebola levels.

### 4. Site-specific parameters

Based upon the data available in the final reports of population-based surveys in each country, model runs for Sierra Leone and Liberia were undertaken at the second administrative unit (representing district-level in Sierra Leone and county-level in Liberia) and at the first administrative unit (regional-level) in Guinea. For each spatial unit, we collated information on parasite prevalence in children under 5 years by microscopy, bed net usage in children under 5 and the proportion of children under 5 with a fever in the past 2 weeks

who had received an anti-malarial. The latter was assumed to be the treatment rate for malaria in each setting. The data are summarised in Table STable S5. To capture the trends in scale-up of bed net coverage (and hence decreasing malaria burden) from 2000 onwards we also extracted bed net usage in previous years and linearly interpolated between these and zero usage in 2000 to obtain annual estimates in scale-up for each spatial unit.

Within the model we take into account the ongoing impact of treatment by including the site-specific proportion of fevers being treated within our baseline scenario, taking into account the proportion of these treatments which were carried out with an ACT (which we assumed provided treatment efficacy and a degree of prophylaxis equivalent to AS-AQ[7]) and those that were provided with non-ACTs (which we assumed provided a similar effect to SP in the absence of substantial SP resistance, with a duration of prophylaxis of approximately 25 days[22]).

Seasonality in transmission was based on an average pattern of rainfall. We used rainfall estimates for Africa from the US Climate Prediction Center [23]. Daily accumulated rainfall since November 2000 is available for a grid of 0.1x0.1 degree resolution. We used the years from 2002 to 2009 and aggregated the daily time series to time series using 64 points per year. Fourier analyses of the data were undertaken in order to capture the seasonality and used to reconstruct the annual rainfall pattern at high resolution using the approach outlined in Garske et al. 2013 [24]. This was aggregated up to the first administrative unit for the analysis presented here.

According to a recent comprehensive world mapping of Anopheles species, all three major African vector species - *An. gambiae s.s., An.arabiensis and An.funestus* – are present to some extent in all three countries [25]. However, *An. gambiae s.s.* and *An funestus* – two highly endophilic and anthropophagic vectors - are dominant in large parts of the region[25], particularly to the south where bed net usage tends to be highest (Table S5). We therefore modelled the vectors as reflecting these species for the entire area using the parameter values reported in Griffin et al. 2010[1].

Population data for each administrative-level was obtained from the most recent census (2004 in Sierra Leone, 2008 in Liberia and 2014 for Guinea). For Sierra Leone and Liberia sub-national estimates were inflated by national-level annual population growth rates[26] to 2014 values and then adjusted to match UN population projection estimates[27].

**Table S5: Summary of data obtained from the most recent DHS or MIS survey**[17–19,28]

| | Parasite prevalence in U5s (% positive on slide microscopy) | Bed net usage in household members (% reporting use the previous night) | Percentage U5s treated with an anti-malarial |
|---|---|---|---|
| **Guinea (survey year)** | **2012** | **2012** | **2012** |
| Boke | 23.5 | 21.2 | 29.1 |
| Conakry | 3.2 | 12.9 | 32.9 |
| Faranah | 66.3 | 26.3 | 34.8 |
| Kankan | 50.1 | 24.6 | 22.1 |
| Kindia | 54.6 | 14.2 | 22.9 |
| Labe | 36.8 | 18.3 | 23.3 |
| Mamou | 46.7 | 12.5 | 15.6 |
| N'Zerekore | 59.2 | 22.1 | 39.2 |
| **Liberia (survey year)** | **2011** | **2013** | **2013** |
| Bomi | 29.0 | 43.9 | 71.2 |
| Bong | 35.0 | 42.9 | 41.8 |
| Gharpolu | 29.0 | 41.2 | 57.4 |
| Grand Bassa | 26.2 | 31.8 | 43.1 |
| Grand Cape Mount | 29.0 | 43.5 | 55.7 |
| Grand Gedeh | 32.6 | 35.2 | 47.2 |
| Grand Kru | 49.2 | 17.5 | 52.5 |
| Lofa | 35.0 | 54.8 | 65.8 |
| Margibi | 26.2 | 31.1 | 64.9 |
| Maryland | 49.2 | 18.0 | 53.3 |
| Montserrado | 26.2 | 24.1 | 49.8 |
| Nimba | 35.0 | 33.8 | 48.5 |
| River Cess | 32.6 | 23.3 | 52.8 |
| River Gee | 49.2 | 36.7 | 64.2 |
| **Sierra Leone (survey year)** | **2013** | **2013** | **2013** |
| Bo | 34.4 | 45.8 | 54.3 |
| Bombali | 51.7 | 45.3 | 41.6 |
| Bonthe | 32.9 | 51.7 | 33.4 |
| Kailahun | 40.4 | 50.8 | 48.1 |
| Kambia | 60.7 | 37.1 | 28.6 |
| Kenema | 37.7 | 50.9 | 50.3 |
| Koinadugu | 55.0 | 34.1 | 22.9 |
| Kono | 57.4 | 33.8 | 37.3 |
| Moyamba | 42.5 | 46.7 | 38 |
| Port Loko | 49.3 | 27.9 | 29.6 |
| Pujehun | 38.9 | 45 | 49.4 |
| Tonkolili | 49.3 | 43.1 | 43.1 |
| Western Area Rural | 35.7 | 31.2 | 68.9 |
| Western Area Urban | 18.6 | 21.7 | 56.3 |

Given these parameters, for each spatial unit we used an iterative process to calibrate our model to these datasets by varying a single parameter – K* - which determines the level of endemicity prior to the scale-up of interventions. Drawing a sample from the joint posterior distribution for the parameters in Table S2 and fixing the suite of other model parameters in Table S3 at their posterior median values, for a proposed value of K* we initially equilibriated the model prior to the year 2000. We then ran the model from 2000 to 2013 including the changing usage of bed nets and for the DHS/MIS reported level of treatment from 2000 onwards to give a model estimate of parasite prevalence in the most recent DHS/MIS survey year. This was repeated with new proposed values of K* until the model estimate of parasite prevalence closely matched the reported value. We repeated this process for 500 draws from the joint posterior distribution of the parameters of the human infection model and with 500 Markov draws from our model of malaria mortality in the presence and absence of a health system to provide posterior distributions of the number of cases and deaths attributable to lack of healthcare due to Ebola.

## 5. Mass drug administration model and scenarios

Mass drug administration was administered monthly in the simulations for a period of either three or six months. At each round, we assume a proportion of individuals in the whole population receive the drug, determined by the coverage. These individuals move into the prophylaxis state P.

The distribution of the duration of prophylaxis was determined by Okell et al. 2014[7] using a PK/PD modelling approach for two artemisinin-combination therapies: AL and DHA-P. Due to the short half-life of the artemisinin component, the duration of protection is determined by the partner drugs in these combinations (lumefantrine and piperaquine respectively). The pharmacokinetic model was based on previous studies and captures age as a proxy for body-weight and the different dosing regimens of the compounds. A combined PK/PD model was fitted to PCR-confirmed re-infection rates in trial data from 6 sites in Africa. This gave an estimate of age-dependent protection over time (see Figure 2 in the original paper) which was input directly into the model for DHA-P. A more recent analysis by Bretscher et al. (2014) has shown no statistically significant differences in the duration of protection from AS/AQ compared to AL[29]. We therefore used the profile for AL as a substitute for AS/AQ in these simulations.

As described in Griffin et al. (2010)[1], we allowed coverage between rounds to be highly correlated, such that those receiving the drug in month 1 also received it in months 2 and 3 whilst those who did not receive it in month 1 did not receive it in months 2 and 3. This represents a conservative scenario as it limits the overall population that receives the drug and is more representative of both geographic and social heterogeneity in distribution and uptake. Each simulation was repeated with 200 draws of the posterior distribution of the malaria transmission model and the assumed ranges in our mortality model to provide credible intervals for the impact of these interventions.

## 6. Comparison of burden estimates with other sources

Health reporting systems in many malaria-endemic countries are insufficient to capture the true burden of malaria disease. Equally, the lack of vital registration data also means that statistics showing absolute population numbers, deaths and causes of death are unreliable.

As a result, global estimates rely on a number of estimation methods. Here we compare the estimates that we obtain to recent estimates made by other groups.

WHO provide estimates of cases and deaths by country in the annual World Malaria Report [9]. Cases are estimated using a risk-based approach in which baseline malaria incidence prior to interventions is defined in 3 age groups (<5, 5-14 and 15+) for a high or low transmission setting (all three countries here being high transmission), the incidence is then reduced by 0.5% for each 1% increase in the proportion of households owning an ITN, and the resulting incidence is multiplied by age-stratified population estimates to obtain case estimates. For deaths, a mortality rate for children under 5 years is estimated from longitudinal studies whilst a mortality rate for those >5 years is derived from a mathematical model of the relationship between the Entomological Inoculation Rate (EIR) and age-specific malaria death rates developed by the Swiss Tropical and Public Health Institute[30]. The death rates are adjusted for ITN use in the same way as for cases. Finally, the under 5 estimates are entered into the Child Health Epidemiology Reference Group model for under 5 mortality as a covariate so that the final estimates of deaths in children under 5 fits within the envelope of all under 5 deaths.

The Malaria Atlas Project (MAP) provided an alternative estimate of malaria cases. Their methodology uses parasite prevalence as measured in surveys as a baseline input. They then used an empirical relationship between parasite prevalence and all-age clinical incidence obtained by fitting to a similar set of data to those used here. Estimates have to date been published for 2007[31]

The Institute for Health Metrics, as part of the Global Burden of Disease study, have also estimated malaria cases and deaths. The approach in this study to estimating both malaria cases and deaths is also risk based. Inputs into the model include the parasite prevalence estimates provided by MAP for 2010, estimated malaria mortality rate, age-group variables and a variable to represent passive versus active case detection. These are fitted to vital registration and verbal autopsy reports globally. As for the WHO estimates, these are constrained to fit within the envelope of overall mortality[32].

Table S6 compares the estimates presented here to the most recent estimates made by these other groups. Our estimates of cases and deaths in Liberia are similar to previous estimates, taking into account population growth. However, in the remaining two countries our estimates are higher than recent WHO estimates (2012) and GBD estimates for 2013 but similar to those estimated by MAP for 2007. Similarly, our estimates of deaths are higher than WHO estimates for 2013 but of similar magnitude to those in GBD for 2013. For both cases and deaths there is considerable uncertainty resulting in overlapping uncertainty intervals across the different group's estimates.

**Table S6: Comparison of estimates of cases and deaths under a functioning health system with recent estimates made by others.**

| | Guinea | Liberia | Sierra Leone | Total | Reference |
|---|---|---|---|---|---|
| **Cases** | | | | | |
| Our estimate for 2015 | 5.3 (3.8-7.2) | 1.6 (1.1-2.2) | 3.4 (2.3-4.6) | 10.9 (8.5-13.3) | |
| MAP 2007 | 4.9 (3.0-6.4) | 2.1 (1.1-2.7) | 3.1 (1.6-4.4) | 10.1 | [31] |
| WHO 2012 | 4.4 (2.3-6.6) | 1.2 (0.6-1.7) | 1.1 (0.6-1.7) | 6.7 | [9] |
| GBD 2013 | 1.9 (1.20, 3.0) | 0.5 (0.3, 0.8) | 1.2 (0.7, 1.8) | 3.6 | [32] |
| **Deaths** | | | | | |
| Our estimate for 2015 | 17,200 (10,300-28,400) | 4,300 (2,700-6,900) | 9,900 (5,900-16,400) | 33,300 (21,800-49,900) | |
| WHO 2012 | 12,000 (9,400-14,000) | 2,900 (2,300-3,500) | 6,500 (4,500-8,300) | 21,400 | [9] |
| GBD 2013 | 14,600 (9,600, 20,500) | 3,200 (1,800, 5,000) | 8,900 (5,700-12,700) | 26,700 | [32] |

One of the most likely reasons for our higher estimates in Guinea is the difference in the underlying prevalence data that we are using to inform the burden estimates. The GBD study uses MAP prevalence estimates from 2010 whilst the WHO method uses a baseline fixed incidence for all high transmission levels. The latter is equivalent to our estimated incidence for parasite prevalence in 2-10 year olds of approximately 20-30%. Table S7 shows the MAP 2010 estimates of parasite prevalence compared to the more recent DHS/MIS surveys used here at the highest available administrative unit for each estimate. Although they do not estimate the same age-range the two estimates are roughly comparable, with the exception of Guinea where 2013 prevalence from DHS, on which our analysis is based, appears substantially higher. Meanwhile, we believe the disparity in our estimates in Sierra Leone is likely to be because ITN usage of 44% as measured in the recent DHS survey is substantially lower than models of ITN availability have previously predicted in that country[9].

**Table S7|Summary of available prevalence estimates in the three countries**

| Region | Map 2010 Prevalence (2-10 year olds) | DHS Prevalence by RDT (0-5 year olds) | DHS Prevalence by Microscopy (0-5 year olds) |
|---|---|---|---|
| **Regions of Guinea (Both at Regional level)** | | | |
| Boké | 0.186106 | 0.367 | 0.235 |
| Conakry | 0.206556 | 0.032 | 0.032 |
| Faranah | 0.411648 | 0.678 | 0.663 |
| Kankan | 0.469968 | 0.647 | 0.501 |
| Kindia | 0.258239 | 0.483 | 0.546 |
| Labé | 0.312437 | 0.318 | 0.368 |
| Mamou | 0.281105 | 0.391 | 0.467 |
| Nzérékoré | 0.507606 | 0.641 | 0.592 |
| **Counties of Liberia (MAP estimate at district level DHS estimate at Regional level)** | | | |
| Bomi | 0.381839 | 0.493 | 0.29 |
| Bong | 0.527953 | 0.495 | 0.35 |
| Gbapolu | 0.451741 | 0.493 | 0.29 |
| Grand Cape Mount | 0.35912 | 0.496 | 0.262 |
| GrandBassa | 0.377121 | 0.493 | 0.29 |
| GrandGedeh | 0.467342 | 0.553 | 0.326 |
| GrandKru | 0.466718 | 0.705 | 0.492 |
| Lofa | 0.492297 | 0.495 | 0.35 |
| Margibi | 0.370426 | 0.496 | 0.262 |
| Maryland | 0.482973 | 0.705 | 0.492 |
| Montserrado | 0.301806 | 0.496 | 0.262 |
| Nimba | 0.534717 | 0.495 | 0.35 |
| River Cess | 0.359357 | 0.553 | 0.326 |
| River Gee | 0.466642 | 0.705 | 0.492 |
| Sinoe | 0.392197 | 0.553 | 0.326 |
| **Districts of Sierra Leone (MAP estimate at regional level DHS estimate at district level)** | | | |
| Bo | 0.471146 | 0.435 | 0.344 |
| Bombali | 0.382461 | 0.532 | 0.517 |
| Bonthe | 0.471146 | 0.304 | 0.329 |
| Kailahun | 0.490787 | 0.416 | 0.404 |
| Kambia | 0.382461 | 0.613 | 0.607 |
| Kenema | 0.490787 | 0.45 | 0.377 |
| Koinadugu | 0.382461 | 0.545 | 0.55 |
| Kono | 0.490787 | 0.514 | 0.574 |
| Moyamba | 0.471146 | 0.491 | 0.425 |
| Port Loko | 0.382461 | 0.519 | 0.493 |
| Pujehun | 0.471146 | 0.351 | 0.389 |
| Tonkolili | 0.382461 | 0.509 | 0.493 |
| Western Area Rural | 0.306161 | 0.477 | 0.357 |
| Western Area Urban | 0.306161 | 0.298 | 0.186 |

**References**


1 Griffin JT, Hollingsworth TD, Okell LC, *et al.* Reducing Plasmodium falciparum malaria transmission in Africa: a model-based evaluation of intervention strategies. *PLoS medicine* 2010; **7**. doi:10.1371/journal.pmed.1000324.

2 Okell LC, Griffin JT, Kleinschmidt I, *et al.* The potential contribution of mass treatment to the control of Plasmodium falciparum malaria. *PloS one* 2011; **6**: e20179.

3 Griffin JT, Ferguson NM, Ghani AC. Estimates of the changing age-burden of Plasmodium falciparum malaria disease in sub-Saharan Africa. *Nature communications* 2014; **5**: 3136.

4 Patil AP, Okiro EA, Gething PW, *et al.* Defining the relationship between Plasmodium falciparum parasite rate and clinical disease: statistical models for disease burden estimation. *Malaria journal* 2009; **8**: 186.

5 WHO Malaria Policy Advisory Committee and Secretariat. Malaria Policy Advisory Committee to the WHO: conclusions and recommendations of March 2013 meeting. *Malaria journal* 2013; **12**: 213.

6 White MT, Griffin JT, Churcher TS, Ferguson NM, Basáñez M-G, Ghani AC. Modelling the impact of vector control interventions on Anopheles gambiae population dynamics. *Parasites & vectors* 2011; **4**: 153.

7 Okell LC, Cairns M, Griffin JT, *et al.* Contrasting benefits of different artemisinin combination therapies as first-line malaria treatments using model-based cost-effectiveness analysis. *Nature communications* 2014; **5**: 5606.

8 United Republic of Tanzania Government. Population and Housing Census. , 2002http://wwwtanzaniagotz/sensa/report2htm.

9 WHO. World Malaria Report 2014. 2014.http://www.who.int/malaria/publications/world_malaria_report_2014/en/ (accessed 6 Feb2014).

10 WHO Malaria Policy Advisory Committee and Secretariat. Malaria Policy Advisory Committee to the WHO: conclusions and recommendations of fifth biannual meeting (March 2014). *Malaria journal* 2014; **13**: 253.

11 Getahun A, Deribe K, Deribew A. Determinants of delay in malaria treatment-seeking behaviour for under-five children in south-west Ethiopia: a case control study. *Malaria journal* 2010; **9**: 320.

12 O'Meara WP, Noor A, Gatakaa H, Tsofa B, McKenzie FE, Marsh K. The impact of primary health care on malaria morbidity--defining access by disease burden. *Tropical medicine & international health : TM & IH* 2009; **14**: 29–35.

13 Dillip A, Hetzel MW, Gosoniu D, *et al.* Socio-cultural factors explaining timely and appropriate use of health facilities for degedege in south-eastern Tanzania. *Malaria journal* 2009; **8**: 144.

14 Reyburn H, Mbatia R, Drakeley C, *et al.* Association of transmission intensity and age with clinical manifestations and case fatality of severe Plasmodium falciparum malaria. *JAMA* 2005; **293**: 1461–70.

15 Von Seidlein L, Olaosebikan R, Hendriksen ICE, *et al.* Predicting the clinical outcome of severe falciparum malaria in african children: findings from a large randomized trial. *Clinical infectious diseases : an official publication of the Infectious Diseases Society of America* 2012; **54**: 1080–90.

16 World Health Organization . World Malaria Report 2008. Geneva, 2008http://www.who.int/malaria/publications/atoz/9789241563697/en/).

17 Ministry of Health and Social Welfare (Liberia), MEASURE DHS. Liberia Demographic and Health Survey 2013. Maryland, U.S.A, 2013 http://dhsprogram.com/pubs/pdf/FR291/FR291.pdf.

18 National Malaria Control Programme (Sierra Leone), MEASURE DHS. Sierra Leone, Malaria Indicator Survey (MIS) 2013. Maryland, U.S.A, 2013 http://dhsprogram.com/pubs/pdf/MIS15/MIS15.pdf.

19 Institut National de la Statistique (Guinee), MEASURE DHS, ICF International. Guinée. Enquête Démographique et de Santé et à Indicateurs Multiples (EDS-MICS) 2012. Maryland, U.S.A, 2013. http://dhsprogram.com/pubs/pdf/FR280/FR280.pdf.

20 Lubell Y, Staedke SG, Greenwood BM, *et al.* Likely health outcomes for untreated acute febrile illness in the tropics in decision and economic models; a Delphi survey. *PloS one* 2011; **6**: e17439.

21 Shillcutt S, Morel C, Goodman C, *et al.* Cost-effectiveness of malaria diagnostic methods in sub-Saharan Africa in an era of combination therapy. *Bulletin of the World Health Organization* 2008; **86**: 101–10.

22 Cairns M, Carneiro I, Milligan P, *et al.* Duration of Protection against Malaria and Anaemia Provided by Intermittent Preventive Treatment in Infants in Navrongo, Ghana. *PLoS ONE* 2008; **3**: 9.

23 National Weather Service . Africa Rainfall Estimates. 2011.http://www.cpc.noaa.gov/products/fews/rfe.shtml .

24 Garske T, Ferguson NM, Ghani AC. Estimating air temperature and its influence on malaria transmission across Africa. *PloS one* 2013; **8**: e56487.

25 Sinka ME, Bangs MJ, Manguin S, *et al.* A global map of dominant malaria vectors. *Parasites vectors* 2012; **5**: 69.

26 Central Intelligence Agency . The World Factbook 2013-14. Washington D.C., 2013https://www.cia.gov/library/publications/the-world-factbook/.

27 Division UNP. World Urbanization Prospects, the 2014 revision. New York City, U.S.A, 2014http://esa.un.org/unpd/wup/CD-ROM/Default.aspx.

28 National Malaria Control Programme (Liberia) ., MEASURE DHS. Liberia Malaria Indicator Survey 2011. Maryland, U.S.A, 2012http://dhsprogram.com/pubs/pdf/MIS12/MIS12.pdf.

29 Bretscher MT, Griffin JT, Hugo P, Baker M, Ghani A, Okell L. A comparison of the duration of post-treatment protection of artemether-lumefantrine, dihydroartemisinin-piperaquine and artesunate-amodiaquine for the treatment of uncomplicated malaria. *Malaria Journal* 2014; **13**: P19.

30 ROSS A, MAIRE N, MOLINEAUX L, SMITH T. AN EPIDEMIOLOGIC MODEL OF SEVERE MORBIDITY AND MORTALITY CAUSED BY PLASMODIUM FALCIPARUM. *Am J Trop Med Hyg* 2006; **75**: 63–73.

31 Hay SI, Okiro EA, Gething PW, *et al.* Estimating the global clinical burden of Plasmodium falciparum malaria in 2007. *PLoS medicine* 2010; **7**: e1000290.

32 Murray CJL, Ortblad KF, Guinovart C, *et al.* Global, regional, and national incidence and mortality for HIV, tuberculosis, and malaria during 1990–2013: a systematic analysis for the Global Burden of Disease Study 2013. *The Lancet* 2014; **384**: 1005–70.
